# Supplementary material for: Progesterone receptor expression contributes to gemcitabine resistance at higher ECM stiffness in breast cancer cell lines
Source: PLoS One. 2022 May 26;17(5):e0268300. doi: 10.1371/journal.pone.0268300 (PMC9135204; doi:10.1371/journal.pone.0268300)

**Supplementary figure 1: Rheological measurements of polyacrylamide hydrogels.**

A representative example of rheological graphs for each gel composition showing elastic modulus, viscous modulus and phase angle. Rheological measurements were made on a Bohlin Gemini 200 rheometer fitted with a 25 mm diameter flat plate at 37 °C. An axial closing force of 0.2-5 N was applied to each hydrogel and the mechanical response was measured over an oscillating frequency range of 10 to 0.01 Hz at a strain of 0.02%. (B) Average elastic modulus ± standard deviation for 3 gels measured over 3 independent repeats for the 5.42% and 7.46% acrylamide gels at a frequency of 1 Hz. (C) Average elastic modulus ± standard deviation for 3 gels measured over 3 independent repeats for the 5.42% and 7.46% acrylamide gels at a frequency of 0.4 Hz.


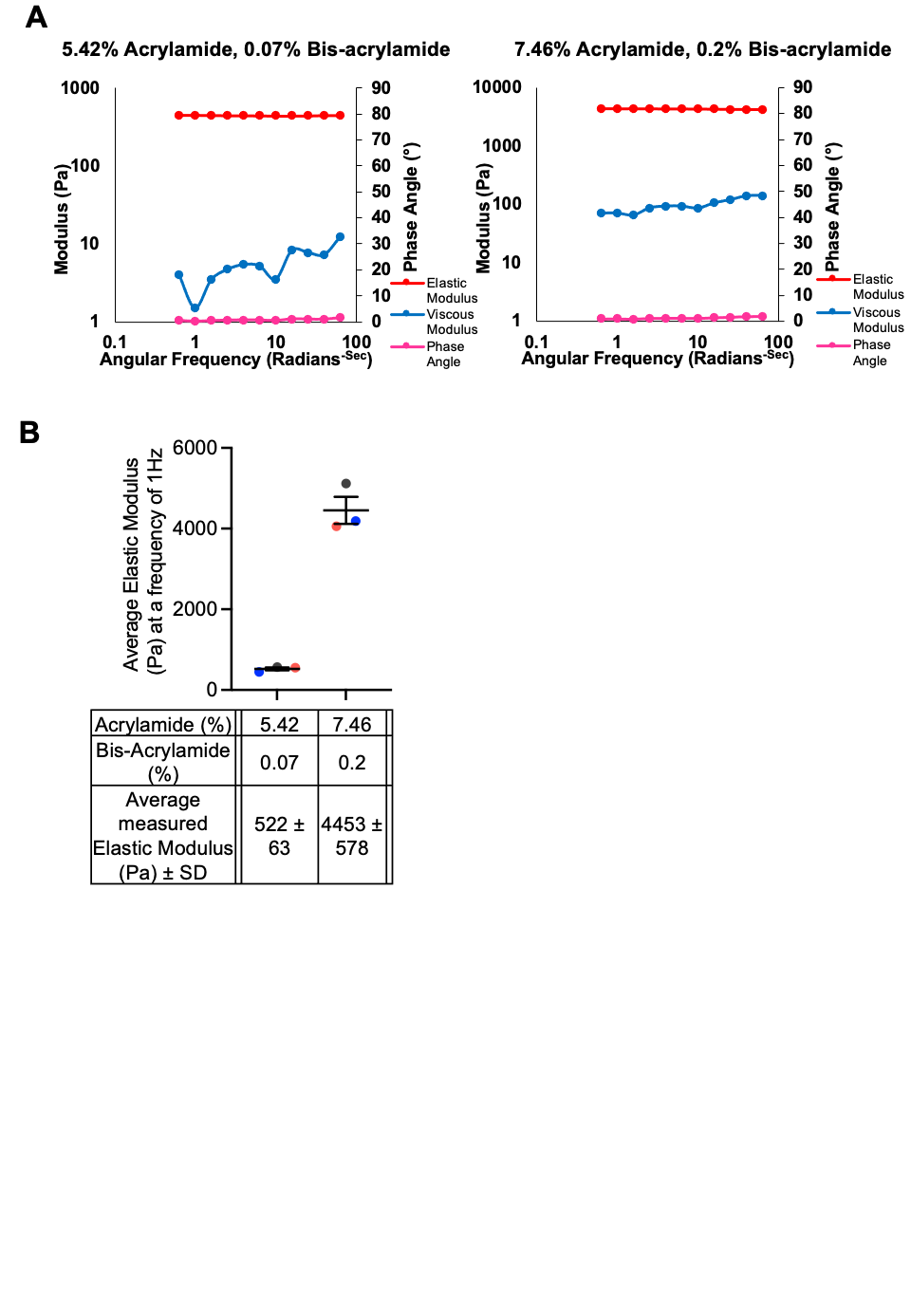

Supplement: S1 Fig — (A) A representative example of rheological graphs for each gel composition showing elastic modulus, viscous modulus and phase angle. Rheological measurements were made on a Bohlin Gemini 200 rheometer fitted with a 25 mm diameter flat plate at 37°C. An axial closing force of 0.2–5 N was applied to each hydrogel and the mechanical response was measured over an oscillating frequency range of 10 to 0.01 Hz at a strain of 0.02%. (B) Average elastic modulus ± standard deviation for 3 gels measured over 3 independent repeats for the 5.42% and 7.46% acrylamide gels at a frequency of 1 Hz. (C) Average elastic modulus ± standard deviation for 3 gels measured over 3 independent repeats for the 5.42% and 7.46% acrylamide gels at a frequency of 0.4 Hz. (DOCX) [file pone.0268300.s001.docx]
