# Supplementary material for: Progesterone receptor expression contributes to gemcitabine resistance at higher ECM stiffness in breast cancer cell lines
Source: PLoS One. 2022 May 26;17(5):e0268300. doi: 10.1371/journal.pone.0268300 (PMC9135204; doi:10.1371/journal.pone.0268300)

**Supplementary figure 2: Rheological measurements of alginate beads.**

A representative example of rheological graphs for each alginate percentage at 1 hr post gelation showing elastic modulus, viscous modulus and phase angle. Rheological measurements were made on a Bohlin Gemini 200 rheometer fitted with a 10 mm diameter flat plate at 37 °C. An axial closing force of 0.2-5 N was applied to each hydrogel and the mechanical response was measured over an oscillating frequency range of 10 to 0.01 Hz at a strain of 0.02%. All gels were made using 200 mM calcium chloride and 4 media washes. (B) Average measured elastic modulus ± standard deviation for 6 gels pooled from 2 independent repeats for the two gel compositions 1 hr after gelation at a frequency of 1 Hz. Each repeat is plotted in a different colour and the mean and standard deviation is plotted in black.


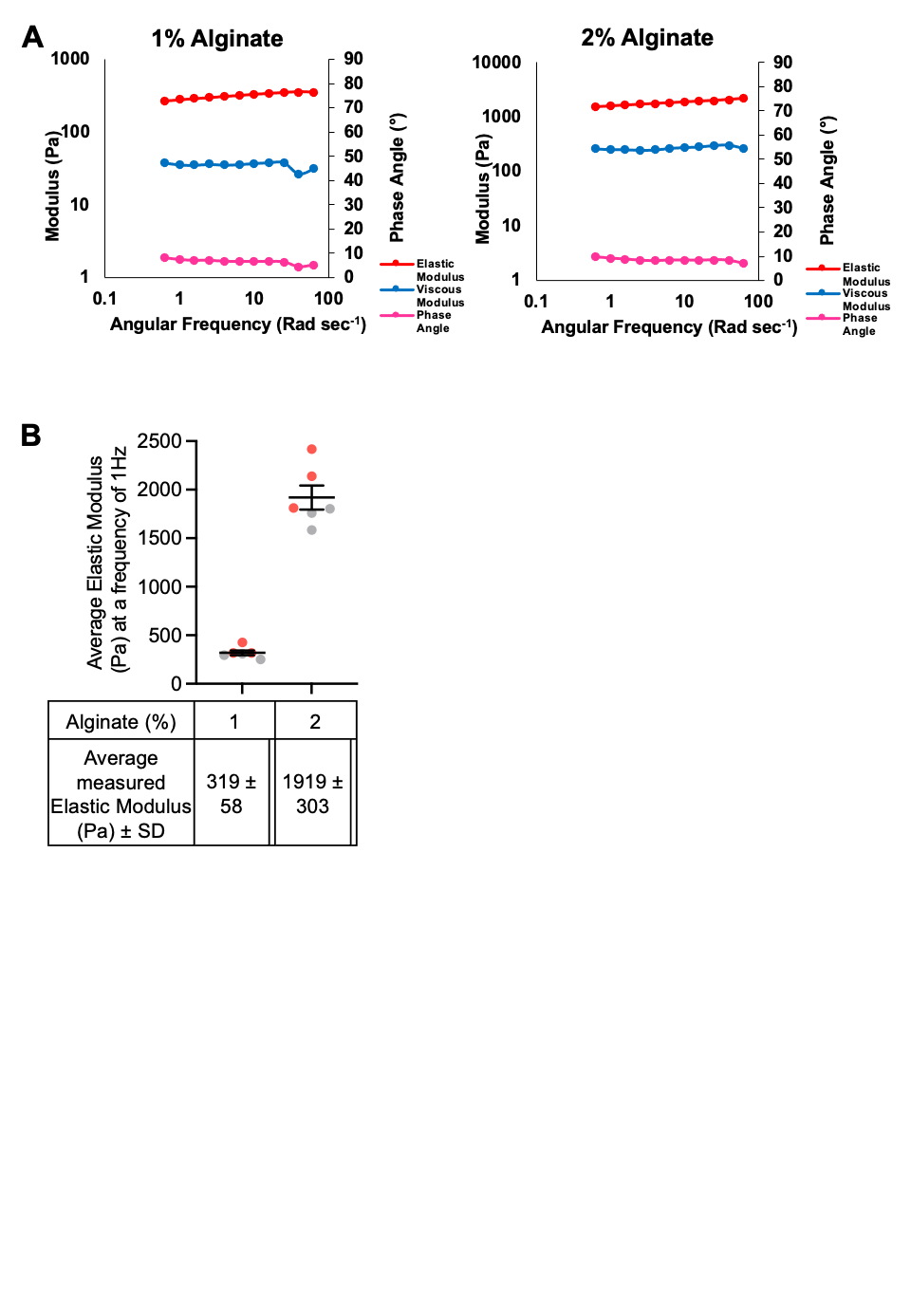

Supplement: S2 Fig — (A) A representative example of rheological graphs for each alginate percentage at 1 hr post gelation showing elastic modulus, viscous modulus and phase angle. Rheological measurements were made on a Bohlin Gemini 200 rheometer fitted with a 10 mm diameter flat plate at 37°C. An axial closing force of 0.2–5 N was applied to each hydrogel and the mechanical response was measured over an oscillating frequency range of 10 to 0.01 Hz at a strain of 0.02%. All gels were made using 200 mM calcium chloride and 4 media washes. (B) Average measured elastic modulus ± standard deviation for 6 gels pooled from 2 independent repeats for the two gel compositions 1 hr after gelation at a frequency of 1 Hz. Each repeat is plotted in a different colour and the mean and standard deviation is plotted in black. (DOCX) [file pone.0268300.s002.docx]
