# Supplementary material for: Progesterone receptor expression contributes to gemcitabine resistance at higher ECM stiffness in breast cancer cell lines
Source: PLoS One. 2022 May 26;17(5):e0268300. doi: 10.1371/journal.pone.0268300 (PMC9135204; doi:10.1371/journal.pone.0268300)
Supplement: S3 Fig — Expression of the total progesterone receptor gene (PGR) and the separate A and B isoforms for MCF-7 cells by q-RT-PCR for one independent repeat. Cells were cultured on 500 Pa or 4 kPa stiffness hydrogels for 72 hrs prior to RNA extraction. Expression of ER total PGR and PGR-B were measured relative to a Glyceraldehyde 3-phosphate dehydrogenase (GAPD) internal control. Fold change in expression was calculated relative to 500 Pa. PGR-A expression was determined by subtracting the fold expression change of PGR-B from that of total PGR expression. (DOCX) [file pone.0268300.s003.docx]

**Supplementary figure 3: RT-PCR analysis of PR and ER expression in cells grown on 500 Pa and 4KPa stiffness gels.**

Expression of the total progesterone receptor gene (*PGR*) and the separate A and B isoforms for MCF-7 cells by q-RT-PCR for one independent repeat. Cells were cultured on 500 Pa or 4 kPa stiffness hydrogels for 72 hrs prior to RNA extraction. Expression of *ER* total *PGR* and *PGR-B* were measured relative to a Glyceraldehyde 3-phosphate dehydrogenase (*GAPD*) internal control. Fold change in expression was calculated relative 500 Pa. *PGR-A* expression was determined by subtracting the fold expression change of *PGR-B* from that of total *PGR* expression.
