# Supplementary material for: Progesterone receptor expression contributes to gemcitabine resistance at higher ECM stiffness in breast cancer cell lines
Source: PLoS One. 2022 May 26;17(5):e0268300. doi: 10.1371/journal.pone.0268300 (PMC9135204; doi:10.1371/journal.pone.0268300)

western blot for E cadherin
p= plastic (excluded from figure 2)
500 = 500Pa
4K = 4kPa

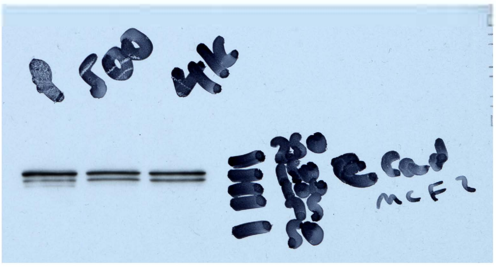

western blot for GAPDH
plastic lane was excluded from figure 2
500 = 500Pa
4K = 4kPa

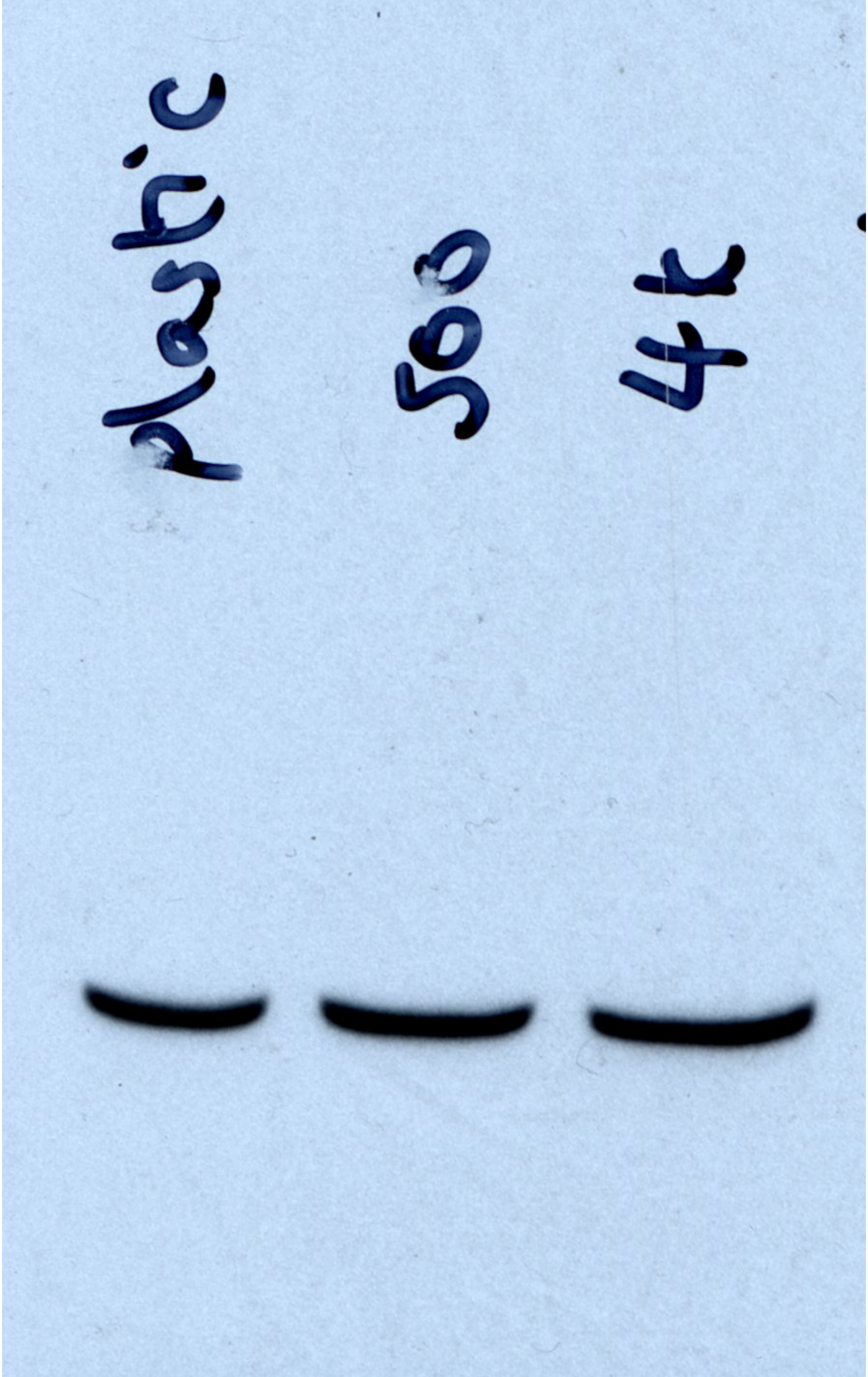

Supplement: S1 Raw images — (PDF) [file pone.0268300.s004.pdf]
